# Progress interrogating TRPM_PZQ_ as the target of praziquantel

**DOI:** 10.1371/journal.pntd.0011929

**Published:** 2024-02-15

**Authors:** Jonathan S. Marchant

**Affiliations:** Department of Cell Biology, Neurobiology & Anatomy, Medical College of Wisconsin, Milwaukee, Wisconsin, United States of America; Walter and Eliza Hall Institute of Medical Research, AUSTRALIA

## Abstract

The drug praziquantel (PZQ) has served as the long-standing drug therapy for treatment of infections caused by parasitic flatworms. These encompass diseases caused by parasitic blood, lung, and liver flukes, as well as various tapeworm infections. Despite a history of clinical usage spanning over 4 decades, the parasite target of PZQ has long resisted identification. However, a flatworm transient receptor potential ion channel from the melastatin subfamily (TRPM_PZQ_) was recently identified as a target for PZQ action. Here, recent experimental progress interrogating TRPM_PZQ_ is evaluated, encompassing biochemical, pharmacological, genetic, and comparative phylogenetic data that highlight the properties of this ion channel. Various lines of evidence that support TRPM_PZQ_ being the therapeutic target of PZQ are presented, together with additional priorities for further research into the mechanism of action of this important clinical drug.

## Introduction

The drug praziquantel (PZQ) has served for decades as the key clinical agent for treating diseases caused by parasitic flatworms. Effective against the majority of these infections [1,2], it is recognized as one of 100 essential medications by the World Health Organization [3]. As a cheap, safe, broadly active, and well-scrutinized clinical therapy, PZQ has served as the keystone of mass drug administration campaigns to decrease the intensity and prevalence of schistosome infections in countries where schistosomiasis is endemic.

**Fig 1:**
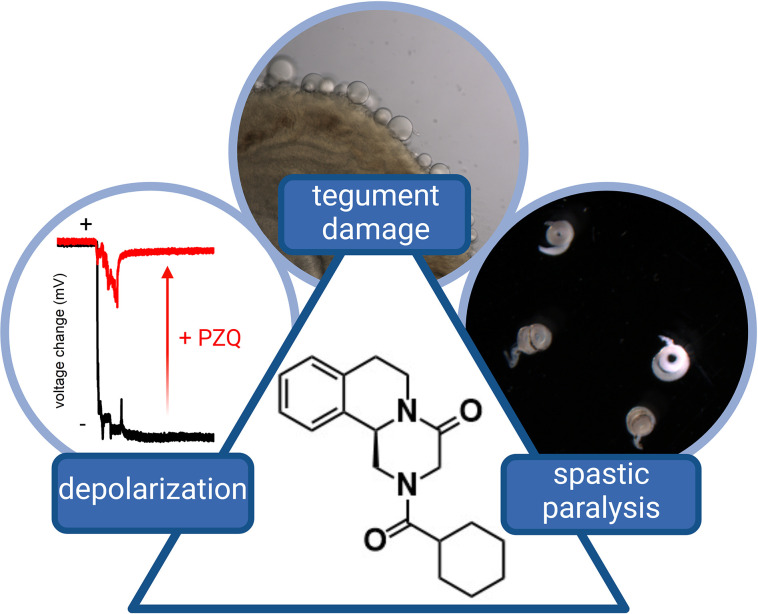
Cardinal effects of PZQ on schistosomes. PZQ treatment of schistosomes is associated with a triad of phenotypic effects: a rapid depolarization of muscle cells, a sustained spastic paralysis of the worm, and broad damage to the tegument manifest as surface blebbing and vesicularization. These effects are most obvious with the (*R*)-enantiomer of PZQ (center). Data are reproduced with permission from [23].

PZQ is, however, an old drug. The anthelmintic activity of PZQ was first realized during a screening collaboration between Merck KGaA and Bayer AG in the 1970s [1,4,5]. Profiling a series of acylated pyrazinoisoquinoline-like compounds revealed the potent activity of PZQ against various trematode and cestodes. Three effects—(i) rapid cellular and tissue depolarization; (ii) a sustained muscle contraction causing worm paralysis; and (iii) damage to the worm tegument apparent as surface “blebbing”—serve as the cardinal triad of features caused by PZQ in all parasitic flatworms where PZQ displays efficacy (**Fig 1**). For each of these effects, the (*R*)-enantiomer of PZQ ((*R*)-PZQ) acted at lower concentrations than the (*S*)-enantiomer ((*S*)-PZQ), evidencing a preference for (*R*)-PZQ at the parasite target. Unfortunately, the identity of this target has resisted definition throughout subsequent decades of clinical usage, placing PZQ within a small minority of FDA-approved drugs with no elaborated molecular target [6].

Nevertheless, PZQ has proved a very effective drug in the clinic. However, opportunities for improvement certainly remain. These include optimization of formulations or derivatives that address the low oral bioavailability and rapid host metabolism of PZQ [7,8], as well as mitigation of other challenges (for example, bitter taste [9]) that result in poor compliance in the field [10]. Further opportunities relate to the lower efficacy of PZQ against certain parasites and life cycle stages—most clearly exemplified by the lack of PZQ activity against *Fasciola* species as well as the poor effectiveness of PZQ against juvenile schistosomes. Our understanding of why PZQ efficacy varies in these situations has been hampered by our lack of knowledge of the molecular target of PZQ. This has long proved a frustrating roadblock. Knowledge of the target would catalyze a better understanding of endogenous signaling pathways essential for parasite viability and thereby vulnerabilities to chemotherapeutic attack. This would also enable target-based drug screening efforts to catalyze discovery of new anthelmintics. Finally, a validated target would enable prospective surveillance for sequence variation, occurring naturally or in response to drug pressure, which could be one of many mechanisms that underpin decreased PZQ effectiveness in the field [11].

For all these reasons, the recent identification of a parasite target for PZQ is a significant breakthrough [12]. This target is a parasite ion channel from the transient receptor potential melastatin family, named TRPM_PZQ_ [12]. TRPM_PZQ_ has been prioritized as an appealing target as it displays properties consistent with the known action of PZQ against parasitic flatworms. The purpose of this review is to summarize experimental evidence collated since the discovery of TRPM_PZQ_ [12,13] that has interrogated the candidature of this ion channel as the clinically relevant target of PZQ. Efforts have focused on understanding (i) the key properties of TRPM_PZQ_; (ii) the impact of variation in TRPM_PZQ_ sequence and expression; and (iii) insight from novel pharmacological tools. Ten pieces of evidence supporting correct target validation of TRPM_PZQ_ are presented, concluding with a discussion of caveats and some future priorities for investigation.

### Key properties of *Sm*.TRPM_PZQ_

In 2019, Park and colleagues identified a TRP channel from *Schistosoma mansoni* (named *Sm*.TRPM_PZQ_) which when heterologously expressed in mammalian cells mediated robust cellular Ca^2+^ signals on exposure to PZQ [12]. Consistent with the long-held focus on a “Ca^2+^ channel hypothesis” for PZQ action [13–15], effort to further investigate the properties of this Ca^2+^-permeable ion channel target held merit.

#### 1. The basic properties of TRPM_PZQ_ replicate the characteristics of PZQ action on schistosomes

Enticingly, the attributes of the TRPM_PZQ_ response to PZQ were consistent with the well-known features of PZQ action on schistosomes. First, the potency of PZQ at TRPM_PZQ_ was in the hundreds of nanomolar range (EC_50_ for (*R*)-PZQ was approximately 150 nM at 37°C), consistent with PZQ action on worms ex vivo [12]. Second, (*R*)-PZQ was more potent than (*S*)-PZQ, consistent with the recognized stereoselectivity of the enantiomers versus parasitic flatworms [12]. Third, the kinetics of activation of TRPM_PZQ_ were rapid in onset with little apparent desensitization of the channel toward PZQ, consistent with the sustained profile of schistosome muscle contraction evoked by PZQ [16,17]. Fourth, the response of *S*. *mansoni* TRPM_PZQ_ to PZQ was attenuated by Mg^2+^ and blocked by La^3+^, consistent with the effects of these metal ions on *S*. *mansoni* muscle contractility [16,17]. Overall, the congruence between these basic characteristics of PZQ-evoked TRPM_PZQ_ activation and worm responsivity to PZQ underscored the promising candidature of TRPM_PZQ_ as the elusive parasitic target of PZQ [12,13].

#### 2. An endogenous current activated by PZQ matches the biophysical signature of TRPM_PZQ_

While identified on the basis of monitoring Ca^2+^ permeability, TRPM_PZQ_ is a nonselective cation channel permeable to both monovalent and divalent cations [18]. Again, this is consistent with the ability of PZQ to stimulate the influx of Na^+^ and Ca^2+^, and the loss of K^+^ from intact schistosomes [19]. Membrane depolarization consequent to TRPM_PZQ_ activation can be resolved in Ca^2+^-free solutions using a fluorescent membrane potential reporter [18] or by recording currents in Ca^2+^-free media [18,20,21]. TRPM_PZQ_ behaves as a voltage-independent ion channel based on a linear current–voltage relationship with a conductance of 110 to 130 pS for *S*. *mansoni*, *S*. *haematobium*, and *S*. *japonicum* TRPM_PZQ_ (recorded in symmetrical 145 mM NaCl) and an open probability, P_open_ = 0.4 to 0.6 [18,21]. These characteristics, combined with a lack of desensitization of channel opening toward PZQ, may endow TRPM_PZQ_ with the ability to mediate a long-lasting depolarization in cell types where it is expressed [18].

However, native PZQ-evoked currents have never been recorded from any parasitic flatworm. This is likely due to the technical challenges of performing these measurements, but also because of our not knowing what exactly to look for and where best to look. Recent effort to resolve the single-channel properties of TRPM_PZQ_ in vitro have established a “biophysical signature” for *Sm*.TRPM_PZQ_, defining a clear search algorithm as well as the optimal recording conditions to search for a native PZQ-evoked current [18]. Further, RNAseq datasets have revealed that TRPM_PZQ_ is expressed in many excitable cells, present in multiple neuronal cell types [22].

These insights improved the feasibility of a new hunt to find an endogenous PZQ-evoked current. Chulkov and colleagues attempted such analyses using invasive electrophysiology to record currents from a live adult schistosome [23]. Despite the challenges of this approach, single-channel responses evoked by PZQ could be resolved from recordings made in “neuronal” tissues, including the anterior ganglia and main nerve cord of male worms [23]. In contrast, no response to PZQ was evident in recordings from “muscle” tissue, or PZQ-derived tegumental vesicles under similar conditions. The native PZQ-activated ion channel displayed properties (linear I-V, Cs^+^ permeability, P_open_, conductance) consistent with the properties of *Sm*.TRPM_PZQ_ measured in vitro [18]. Further, the PZQ-evoked current was blocked by a *Sm*.TRPM_PZQ_ antagonist [23]. That the properties of an endogenous PZQ-activated current in an adult schistosome closely match the characteristics of *Sm*.TRPM_PZQ_ support correct target identification.

#### 3. TRPM_PZQ_ is present in all flatworms that show sensitivity to PZQ

TRPM_PZQ_ must be present in all parasites that exhibit sensitivity to PZQ; otherwise, another target must exist to mediate PZQ action in these worms. Bioinformatic analyses have shown this to be the case. Scrutiny of available genomic and transcriptomic resources revealed the revealed the presence of TRPM_PZQ_ orthologs in all available flatworm genomes [12,24]. TRPM_PZQ_ orthologs from 11 of these different species have been functionally profiled in vitro. These encompass TRPM_PZQ_ from schistosomes (*S*. *mansoni*, *Sm*.TRPM_PZQ_; *S*. *japonicum Sj*.TRPM_PZQ_; and *S*. *haematobium*, *Sh*.TRPM_PZQ_), *Clonorchis sinensis* (*Cs*.TRPM_PZQ_), *Opisthorchis viverrini* (*Ov*.TRPM_PZQ_), *Echinostoma caproni* (*Ec*.TRPM_PZQ_), *Fasciola* species (*F*. *hepatica*, *Fh*.TRPM_PZQ_ and *F*. *gigantica*, *Fg*.TRPM_PZQ_), tapeworms *(Echinococcus granulosus*, *Eg*.TRPM_PZQ_ and *Mesocestoides corti*, *Mc*.TRPM_PZQ_) as well as a free-living flatworm representative (*Macrostomum lignano*, *Ml*.TRPM_PZQ_). All these orthologs, with the exception of TRPM_PZQ_ from *Fasciola* species (*Fh*.TRPM_PZQ_ and *Fg*.TRPM_PZQ_) are sensitive to PZQ, with (*R*)-PZQ being the more active enantiomer in every case [24,25]. While not a comprehensive analysis, data from these functional profiling efforts to date remain consistent with the known clinical utility of PZQ for treating infections caused by these different parasitic flatworms. *Fasciola* infections are known to be refractory to PZQ treatment. Our understanding of the molecular basis for this insensitivity is discussed in the next section.

### Genetic variation impacting TRPMPZQ function

Additional support for TRPM_PZQ_ as the therapeutically relevant target of PZQ comes from the clear correlation between species- and strain-specific properties of TRPM_PZQ_ and the overall sensitivity of these different parasitic flatworms to PZQ.

#### 4. *Fasciola* TRPM_PZQ_ provides a clear molecular explanation for the insensitivity of these liver flukes to PZQ

Liver flukes from the genus *Fasciola* are insensitive to PZQ and epsiprantel [2,26,27]. Human fascioliasis is refractory to treatment by PZQ [28,29]. An explanation for the lack of PZQ efficacy against these particular parasites has long been lacking. Suggestions have encompassed an impermeability of the liver fluke tegument to PZQ, efficient export of PZQ from *Fasciola*, or that the target of PZQ is absent in *Fasciola* spp. [26]. TRPM_PZQ_ is, however, present in *Fasciola* spp. (see point #3), and functional analysis of TRPM_PZQ_ provided a simple explanation for why PZQ does not work against these particular liver fluke infections.

**Fig 2:**
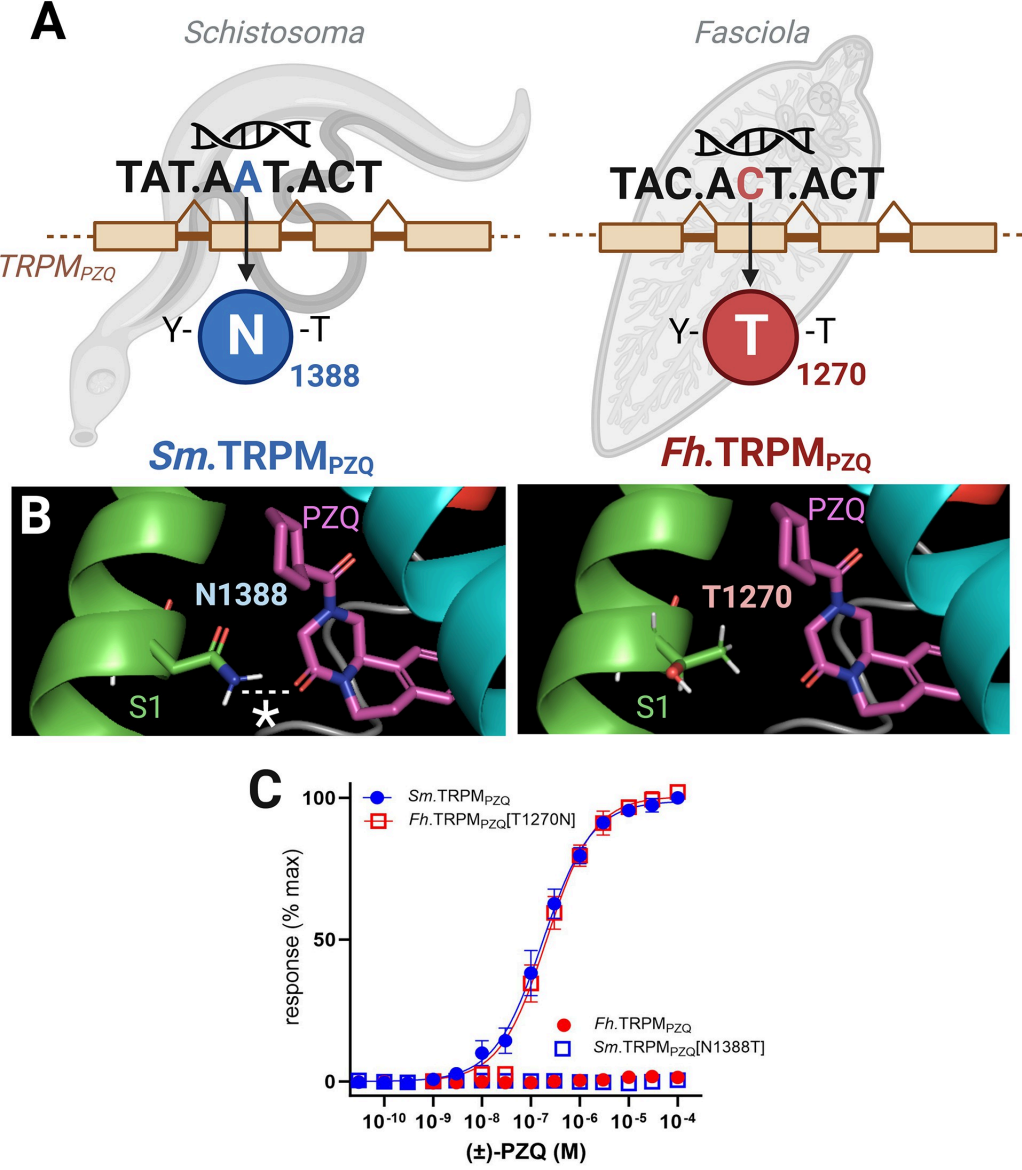
Genetic determinants of PZQ sensitivity. (**A**) A single nucleotide difference occurs at position 2 of the reading frame within an exon of the *TRPM*_*PZQ*_ gene that forms part of the PZQ binding pocket in *S*. *mansoni TRPM*_*PZQ*_ (left) and *F*. *hepatica TRPM*_*PZQ*_ (right). This results in different amino acids—asparagine in *S*. *mansoni* TRPM_PZQ_ (N1388 in *Sm*.TRPM_PZQ_) versus threonine in *F*. *hepatica* TRPM_PZQ_ (T1270 in *Fh*.TRPM_PZQ_)—at an equivalent position within the VSLD binding pocket in TRPM_PZQ_ of these different flukes. This difference also occurs in *F*. *gigantica TRPM*_*PZQ*_. (**B**) Location of this S1 helix reside (N1388 in *Sm*.TRPM_PZQ_ versus T1270 in *Fh*.TRPM_PZQ_) in a homology model *Sm*.TRPM_PZQ_ relative to the predicted PZQ binding poise (magenta). The availability of N1388 to hydrogen bond with the internal carbonyl group of PZQ is inferred as important for PZQ activation of *Sm*.TRPM_PZQ_. T1270 is either unavailable for hydrogen bonding, or this variation impacts binding pocket architecture in a manner deleterious to PZQ efficacy. (**C**) Concentration response curves comparing activation of wild-type *Sm*.TRPM_PZQ_ and *Fh*.TRPM_PZQ_ by PZQ (circles), as well as the effect of the reciprocal binding pocket mutants (*Sm*.TRPM[N1388T]_PZQ_ and *Fh*.TRPM[T1270N]_PZQ_, squares) on responsivity to (±)-PZQ. Data adapted from [25]. Panels in this Figure were created using BioRender.com.

Park and colleagues identified a single nucleotide variation between *Fasciola* TRPM_PZQ_ and TRPM_PZQ_ in other trematodes that yields an amino acid change within the binding pocket of *Fasciola* TRPM_PZQ_, encoding a threonine residue instead of an asparagine residue (**Fig 2A**; [25]). This difference occurs at a critical position that is necessary for binding PZQ: The asparagine residue in transmembrane helix 1 (TM1) of schistosome TRPM_PZQ_ is predicted to form a hydrogen bond with the internal carbonyl of PZQ [25] (**Fig 2B**). This interaction is predicted to be lacking between PZQ and *Fasciola* sp. TRPM_PZQ_ (**Fig 2C**). When this residue was mutated to a threonine in *Sm*.TRPM_PZQ_, PZQ could no longer activate the ion channel [25]. Reciprocally, mutation of the threonine residue to an asparagine within *Fasciola hepatica* or *Fasciola gigantica* TRPM_PZQ_ realized a “gain-of-function,” and PZQ became a potent activator of the *Fasciola* TRPM_PZQ_ channel [24,25] (**Fig 2D**). Therefore, even though this variation represents a minimal and conservative amino acid replacement, the change in the TRPM_PZQ_ binding pocket was sufficient to abrogate PZQ activity [24,25].

The selective pressures, if any, underpinning this change in *Fasciola* TRPM_PZQ_ are unknown. Possibly, it may relate to the exposure to natural products during the *Fasciola* lifecycle (for example, compounds in watercress leaves where infective metacercariae are attached [30]) that could adversely activate TRPM_PZQ_ in the absence of such this adaptation within the ligand binding pocket. Many phytochemicals act as TRP channel ligands [31].

Whatever the explanation, elucidation of the molecular basis of *Fasciola* TRPM_PZQ_ insensitivity toward PZQ enabled a rational approach to develop new fasciocidal agents that are tolerant of this variation. Development of novel TRPM_PZQ_ activators is currently a focus of ongoing investigation. One such chemotype—a benzamidoquinazolinone (BZQ)—which potently activated both *Sm*.TRPM_PZQ_ and *Fh*.TRPM_PZQ_ was recently identified [32]. The basis for this dual activation depends on a different binding conformation of BZQ within the TRPM_PZQ_ VSLD binding pocket, such that the variant position on the S1 helix is not important for BZQ binding [32]. BZQ engages the S1 helix through a different interaction, conserved in both *Sm*.TRPM_PZQ_ and *Fh*.TRPM_PZQ_. Exposure of schistosomes to BZQ, like PZQ, caused a rapid and sustained contraction with obvious surface damage. Similarly, application of BZQ to *Fasciola hepatica* also caused a rapid, spastic contraction and tegumental damage. That a *Fasciola* TRPM_PZQ_ activator identified by target-based screening is deleterious to liver fluke and phenocopies PZQ action on schistosomes further supports correct target validation of TRPM_PZQ_.

This molecular insight should prompt wariness given the precedence this explanation establishes for the viability of a PZQ-insensitive TRPM_PZQ_ channel maintained over evolutionary time. It would obviously be concerning if a similar TRPM_PZQ_ variant was found naturally or occurred in response to the selective pressure associated with PZQ exposure during mass drug administrative campaigns. Analysis of TRPM_PZQ_ sequence and/or expression levels as potential routes to PZQ resistance would be worthwhile in scenarios such as persistent “hotspots” after mass drug administration campaigns [33] or obvious losses of PZQ efficacy in veterinary [34,35] or aquaculture treatments [36,37]. Effort to catalogue TRPM_PZQ_ variants, coupled with insight as to the functional consequences of such variation, will be crucial for surveillance of changes underpinning decreases in the clinical effectiveness of PZQ.

#### 5. Schistosomes genetically selected for low PZQ sensitivity show low expression of TRPM_PZQ_

A genome-wide association study using a mixed population of PZQ-resistant and PZQ-sensitive *S*. *mansoni* worms identified a 4MB region on chromosome 3, which harbored many genes at the highest association peak, including the gene encoding *Sm*.TRPMPZQ [38]. Marker-assisted selection using a single nucleotide polymorphism (SNP) present in the *Sm*.TRPMPZQ gene and associated with PZQ responsiveness allowed for the generation of 2 populations of schistosomes that displayed a remarkable >377-fold difference in PZQ sensitivity. These populations were fixed for alternative alleles at a SNP genotyped within *Sm*.TRPM_PZQ_, as well as 2 different proximal 150 kb deletions (one quite close to *Sm*.TRPM_PZQ_ and one near a transcription factor). Of the genes within this locus, *Sm*.TRPM_PZQ_ uniquely exhibited lower expression in adult male worms that displayed poor PZQ sensitivity [38]. This elegant body of work provides strong evidence that variation within, or near to, the *TRPM*_*PZQ*_ locus confers decreased responsivity to PZQ [38,39].

One explanation for this observation is that the lower sensitivity to PZQ results from lower levels of *Sm*.TRPM_PZQ_ expression, which results in a less robust response or a more facile recovery of cells after PZQ exposure. That differential expression of *Sm*.TRPM_PZQ_ may result in varied sensitivity to PZQ also chimes with the stronger response to PZQ in adult male versus female schistosomes [40,41], which correlates with the higher levels of *Sm*.TRPM_PZQ_ expression in male worms [38,42]. *Sm*.TRPM_PZQ_ shows lower expression in male or female worms that display low sensitivity to PZQ [38]. However, TRPM_PZQ_ mRNA levels in juvenile schistosomes are equivalent or higher than those found in adults [38,42], so other explanations must underpin the lower sensitivity of immature worms to PZQ. Again, this may be caused by a less robust response (unique regulation of TRPM_PZQ_ activity or ion channel expression in this specific lifecycle stage) or a more facile recovery to drug exposure (stronger tissue repair in juvenile worms). Overall, resolution of the regulatory mechanisms that control TRPM_PZQ_ expression and correlating TRPM_PZQ_ expression in parasitic flatworms with the effectiveness of PZQ treatment will be priorities for future study.

#### 6. The differential sensitivity of various parasites to PZQ correlates with the differential PZQ sensitivity of TRPM_PZQ_ orthologs in these different flatworms

The sensitivity of different parasitic flatworms toward PZQ is not the same; for example, while PZQ was originally recognized as effective against trematodes and cestodes [1,43], the sensitivity of cestodes to PZQ is highest. Sensitivity to PZQ is manifest at low nanomolar concentrations against some cestodes [44,45]. Blood flukes typically respond to PZQ in the hundreds of nanomolar range [2], and free-living flatworms show responses in the micromolar range [24]. *Fasciola* spp. represent an extreme example showing lack of sensitivity toward PZQ.

Efforts to measure the sensitivity of TRPM_PZQ_ orthologs from representatives of each of these groups demonstrated that TRPM_PZQ_ sensitivity to PZQ aligns well with the observed worm sensitivity to PZQ. Two cyclophyllidean cestode TRPM_PZQ_ channels—*Mesocestoides corti* TRPM_PZQ_ (*Mc*.TRPM_PZQ_) and *Echinococcus granulosus* TRPM_PZQ_ (*Eg*.TRPM_PZQ_)—were potently activated by (*R*)-PZQ (EC_50_ = 82 ± 3 nM for *Mc*.TRPM_PZQ_, EC_50_ = 55 ± 6 nM for *Eg*.TRPM_PZQ_; [24]), consistent with the high sensitivity of this group of cestodes to PZQ. In contrast, TRPM_PZQ_ from a free-living flatworm representative, *Macrostomum lignano* (*Ml*.TRPM_PZQ_), was activated by (*R*)-PZQ with approximately 300-fold lower potency (EC_50_ = 18 ± 0.8 μM; [24]), consistent with the concentration range of PZQ activity against free-living flatworms. Variation in the amino acids residues that line the orthosteric binding pocket of the different TRPM_PZQ_ orthologs likely contributes to this differential sensitivity. Functionally impactful residues include (i) a histidine residue in cestode TRPM_PZQ_ orthologs found at the same S1 helix position, which dictates the PZQ insensitivity of *Fasciola*, and (ii) an acidic amino acid residue found within the TRP helix. This latter residue, represented by an aspartic acid residue in the high sensitivity TRPM_PZQ_ orthologs of trematodes and cyclophyllidean cestodes, is a glutamic acid residue in other parasitic TRPM_PZQ_ orthologs and TRPM paralogs [24]. The presence of the glutamic acid variant confers lower sensitivity to PZQ in these channels, with application of molecular dynamics and metadynamic modelling methods suggesting the glutamic acid residue projects into the PZQ binding pocket ablating a critical receptor–ligand interaction required for high affinity PZQ binding [24]. Identification of this acidic “gatekeeper” residue provides an explanation for the lower observed clinical sensitivity to PZQ in parasitic flatworm infections that harbor a glutamate at this position, while parasites that carry an aspartate at this position (trematodes and cyclophyllidean cestodes) uniquely display high sensitivity to PZQ [2,46]. The properties of TRPM_PZQ_ orthologs from different flatworms, therefore, show a consistent correlation between worm sensitivity to PZQ and TRPM_PZQ_ ortholog sensitivity to PZQ.

### Pharmacology of TRPM_PZQ_

With a candidate parasite target for PZQ identified, target-based drug screening approaches become feasible. Such efforts have realized additional insight into the properties of TRPM_PZQ_.

#### 7. The structural–activity relationship of drugs causing *Sm*.TRPM_PZQ_ activation and worm contraction are similar

Functional profiling of a series of 43 PZQ analogs and nonobvious derivatives against *Sm*.TRPM_PZQ_ ranked the potency of all these analogs in terms of TRPM_PZQ_ activation [25]. The same analogs were then examined for their potency in causing spastic contraction of adult schistosome worms [25]. The structure–activity fingerprint for these analogs was almost identical in both assays. The “tightness” of the structure–activity relationship (SAR) of PZQ analogs at causing paralysis was also mirrored by the same strict SAR for efficacy at *Sm*.TRPM_PZQ_. Such stringency around the pharmacophore of PZQ has long been recognized [1]. Retrospective analysis of data from over 250 PZQ analogs revealed only 4% of synthesized PZQ derivatives displayed equivalent activity to PZQ [47]. Such congruence between the SAR of the contractile response and the pharmacological profile of this ion channel in vitro is again consistent with *Sm*.TRPM_PZQ_ acting as the mediator of PZQ action.

#### 8. The different structure–activity relationship of TRPM_PZQ_ between parasitic flatworms varies in line with worm sensitivity to different analogs

When the SAR of PZQ was elaborated [1], it became evident that particular PZQ analogs displayed differential activities against different types of parasite. For example, some PZQ analogs—3-pyridine analogs and certain modifications of the cyclohexyl ring—showed considerably greater activity toward cestodes than against schistosomes [1]. Is this differential bioactivity mirrored by different structure activity relationships at schistosome and cestode TRPM_PZQ_? The answer is yes, with good alignment between analog activity against different parasites and the underlying selectivity of TRPM_PZQ_ orthologs in these different species [48]. The SAR of TRPM_PZQ_ in different parasites is, therefore, not identical. Differences in the amino acids lining the transmembrane TRPM_PZQ_ ligand binding pocket likely underpin these effects, highlighting future potential for developing drugs tailored toward specific TRPM_PZQ_ targets and problematic clinical infections that are more refractory toward PZQ treatment. That the distinct pharmacological signatures of parasites toward PZQ analogs mirrors the properties of TRPM_PZQ_ in the different parasites further supports TRPM_PZQ_ as the therapeutically relevant target.

#### 9. Other agonists of TRPM_PZQ_ phenocopy PZQ. If TRPM_PZQ_ is the target of PZQ, other activators of TRPM_PZQ_ should mimic PZQ action

If such TRPM_PZQ_ activators do not phenocopy PZQ action, then PZQ must also engage other targets that contribute to the drug’s therapeutic activity. To develop this line of enquiry, alternative activators of TRPM_PZQ_ needed to be found. Chulkov and colleagues executed a target-based screen of approximately 16,000 compounds against *Sm*.TRPM_PZQ_ using a miniaturized fluorescence reporter assay [49]. This screen resulted in the identification of a single TRPM_PZQ_ agonist “hit” that surpassed triage criteria. This low hit rate (0.06%) in this screen was again consistent with the known stringent SAR of PZQ and TRPM_PZQ_. The *Sm*.TRPM_PZQ_ activator was named AG1 (agonist-1) and was less potent than PZQ (EC_50_ approximately 9 μM; [49]). Nevertheless, AG1 activated *Sm*.TRPM_PZQ_ similarly to PZQ, eliciting non-desensitizing, ohmic currents when profiled electrophysiologically [49]. Further, a VSLD binding pocket mutant ablated both PZQ and AG1 activation of *Sm*.TRPM_PZQ_, suggesting a similar action of both ligands through engagement of the transmembrane VSLD ligand binding site. While this does not prove that AG1 is selective for *Sm*.TRPM_PZQ_, a novel *Sm*.TRPM_PZQ_ activator was found from this screening effort. Notably, AG1 represented a different chemotype to PZQ. Whereas PZQ has a pyrazinoisoquinoline core, AG1 is a triazolopyridine derivative. Their common activation of TRPM_PZQ_ highlighted the druggability of this novel target. Interestingly, AG1 was a known compound (MV688313, LSHTM-1945) previously identified as a high priority “hit” in a large, phenotypic screen (approximately 300,000 compounds) against different schistosome life cycle stages [50]. That a phenotypic screen and a target-based screen completed by independent groups converged on the same ligand, unmasked as a *Sm*.TRPM_PZQ_ activator, provided further reassurance of correct target identification.

#### 10. Antagonists of *Sm*.TRPM_PZQ_ decrease parasite sensitivity toward PZQ

The same target-based screen against *Sm*.TRPM_PZQ_ also yielded many potential blockers of this channel [49]. These candidate blockers have yet to be studied and appraised as to their mode of action, for example, whether they function as competitive inhibitors of PZQ within the same VSLD binding pocket, pore blockers, or noncompetitive inhibitors of the TRPM_PZQ_ complex. Only the effects of one compound, ANT1 (“antagonist-1”), have been evaluated to date. ANT1, a substituted pyrazine, blocked the effects of PZQ measured in either a metabolic or a motility assay, such that ANT1 application recovered normal worm movement and viability in the presence of PZQ [38,49]. That a *Sm*.TRPM_PZQ_ antagonist blocks the action of PZQ on schistosomes again supports the candidacy of TRPM_PZQ_ as the relevant in vivo target of PZQ. ANT1 also blocked the native current evoked by PZQ in schistosomes [23]. The utility of TRPM_PZQ_ blockers (as opposed to TRPM_PZQ_ activators) as potential anthelmintics has not yet been explored. However, further study of these chemotypes is important as these efforts will provide useful tools for inhibiting the function of TRPM_PZQ_ to unmask the endogenous role of this ion channel throughout the parasite lifecycle.

### Caveats and future directions

Collectively, these 10 lines of evidence provide strong support for TRPM_PZQ_ acting as the therapeutic target of PZQ, with the experimental data discussed above proving consistent with correct target validation. However, caution is always merited, and further questions remain. One wryly notes that even for cancer drugs undergoing clinical trials in humans, their assumed targets have often retrospectively been shown to be false [51,52]. This underscores the importance of coalescing multiple lines of evidence to underpin target validation [52,53]. In this regard, 3 areas merit further attention [13].

#### Validation through genetic loss of function analyses

Insight from functional genetic approaches is needed. Results from knockdown or knockout analyses, to ablate TRPM_PZQ_ expression in parasites, have yet to be reported. Neither of these approaches are trivial to execute: Knockdown by RNA interference (RNAi) can be finicky depending on the target, how abundant it is and where it is expressed in the worm. Stable transgenesis in schistosomes is also an active focus for optimization. TRPM_PZQ_ is not abundantly expressed at the surface of the worm but is found within excitable cells. The large cation flux mediated by TRPM_PZQ_ would likely necessitate a highly penetrant knockdown of TRPM_PZQ_ for RNAi data to be interpretable, as residual expression of TRPM_PZQ_ could still support a robust depolarization response to PZQ. Challenges related to off-target effects with RNAi, and the adequacy of controls for many commonly scored phenotypes, also persist [54]. But provided TRPM_PZQ_ is not crucial for parasite viability, these genetic loss-of-function approaches will provide critical insight as to the essentiality of TRPM_PZQ_ for PZQ action. The availability of small molecule blockers of TRPM_PZQ_ (see point #10) will complement these genetic loss-of-function approaches as pharmacological blockade of TRPM_PZQ_ should phenocopy and thereby validate RNAi effects. Clearly, if PZQ-evoked depolarization, contraction, and surface damage phenotypes persist in the absence of TRPM_PZQ_, then other targets must mediate these effects. TRPM_PZQ_, despite the aforementioned evidence, would then be a “false” target in relation to the anthelmintic activity of PZQ.

#### Other targets?

PZQ inevitably has more than one target, consistent with the polypharmacological profile expected with any small molecule [55,56]. Many of these will be “secondary” targets, with these interactions not recapitulating the high sensitivity and stereoselectivity displayed by TRPM_PZQ_ (the “primary” target). For example, in humans, where the process of target identification is more facile, PZQ has been shown to regulate multiple TRP channels [57,58] and several GPCRs [59]. However, these interactions exhibit lower sensitivities (micromolar at best) and often different stereochemistry (for example, hTRPM8 is only activated by (*S*)-PZQ [58]). For the human 5-HT_2B_ receptor, where a (*R*)-PZQ binding pose has been defined and validated, the lower sensitivity of 5-HT_2B_ toward PZQ (EC_50_ in low micromolar range [59,60]) can be explained by the loss of specific binding interactions that been shown to anchor PZQ within the schistosome TRPM_PZQ_ binding pocket. For example, whereas hydrogen-bonding interactions occur in TRPM_PZQ_ to both the carbonyl groups of PZQ, only a single hydrogen-bond interaction is predicted in the human 5-HT_2B_ binding pocket [60]. Loss of optimal hydrogen-bond interactions will decrease binding affinity [61], likely explaining the shift from the “hundreds of nanomolar” to the “micromolar” sensitivity range, even though “selective” binding (5-HT_2B_ compared with 5-HT_2A_ or 5-HT_2C_) is still evident. These host targets may be relevant to several side effects associated with PZQ (5-HT_2B_: smooth muscle contraction underpinning nausea, abdominal pains; TRPM8: poor taste), and, potentially, also therapeutic efficacy (vascular contraction in mesenteric vessels) by aiding the hepatic shift of contracted worms [59,60].

Just as with the discovery of such “secondary” targets in humans, secondary parasite targets for PZQ will be discovered. Indeed, several PZQ-interacting proteins in schistosomes have already been proposed including myosin light chain [62], actin [62,63] (but see [64]), voltage-operated Ca^2+^ channels [65], multidrug-resistant transporters [66], adenosine transporters [67], glutathione S-transferase (GST) [68], and several members of the tegumental allergen (TAL) family of proteins [69]. However, for the majority of these candidates, quantitative characterization of PZQ binding and the selectivity of the ligand binding site is lacking. Also, acknowledging the tight SAR of PZQ and the reciprocally tight SAR of the TRPM_PZQ_ binding pocket, it is worth pointing out that many conjugated PZQ analogs utilized in prior target discovery strategies would poorly interact with TRPM_PZQ_, if at all. Whether any of these reported interactions contribute to the therapeutic efficacy of PZQ remains the critical question, and this will require careful validation. Three fundamental criteria, outlined in the preceding sections for TRPM_PZQ_, must be met. First, is there reasonable congruence between the affinity for PZQ at the proposed target versus PZQ efficacy against the worm? Second, is there a similar SAR for PZQ analogs at the proposed target versus the parasite? Third, is there a clear functional outcome consequent to PZQ engaging these targets that is consistent with the triad of phenotypic outcomes (depolarization, worm contraction, tegument damage)? For example, with *Sm*.TAL1, where careful efforts have been made to characterize PZQ binding, the resolved affinity is low (K_d_ of *Sm*.TAL1 for PZQ = 140 μM [69]) compared with PZQ action on worms. For schistosome GST, where PZQ was cocrystallized with the enzyme, the binding site lies within an amphipathic groove at the dimer interface, which promiscuously accommodates many hydrophobic chemotypes [21] not reflecting the established SAR. Further, PZQ binding does not affect GST function [70]. Therefore, many of these proposed interactors may not stand up to scrutiny as a “primary” target.

Could there be another “primary” parasite target that matches the sensitivity, SAR, and functional impact of TRPM_PZQ_? Another “primary” target cannot be excluded, while so many ion channels and GPCRs remain unprofiled in parasitic flatworms. However, one notes the insensitivity of *Fasciola* toward PZQ: If there is another “primary” target for PZQ, then the effects of engagement of this target should be manifest in *Fasciola* in the absence of any contribution from TRPM_PZQ_. However, *Fasciola* spp. remain insensitive to very high (millimolar) concentrations of PZQ [26], consistent with a single “primary” target model. Notwithstanding this argument, the most likely place to look for additional PZQ targets might be other TRP (and likely TRPM) family members, as many of these channels have yet to be studied. This is a challenge in the absence of known activators to confirm successful heterologous expression of each ion channel candidate, as a negative result is not definitive in the absence of a positive control. However, a second TRPM family member has recently been deorphanized and shown to respond to the benzodiazepine, meclonazepam, an old anthelmintic [71]. This channel, named TRPM_MCLZ_, did not respond to PZQ as predicted [24,71]. However, the fact that TRPM_MCLZ_—a cousin of TRPM_PZQ_—also mediates worm contraction, depolarization, and surface damage is broadly supportive of functional assignment of both anthelmintic targets to the same ion channel subfamily. This underscores the promise of TRPM channels for design and development of new anthelmintics.

#### What has it got in its pocketses?

It is fair to ask—what endogenous ligands activate TRPM_PZQ_? TRPM_PZQ_ is a large channel, with each monomer composed of approximately 2,200 amino acids (predicted as approximately 250 kDa). The expected tetramer would be of an exceptionally large size (>1 MDa). Within this quaternary structure, there are likely a multitude of nooks and crannies that could accommodate ligands, lipids, and accessory proteins. For example, as many as 16 unique “ligand” binding sites have been counted in human TRPV channels [72], many of which are conserved in other TRP family members [73,74]. Only the VSLD binding pocket has been mapped so far in TRPM_PZQ_. Therefore, there is much work to do: This encompasses provision of a structural solution to TRPM_PZQ_ architecture, identification of TRPM_PZQ_ regulators, and then definition of their role in regulating TRPM_PZQ_ responsiveness.

Already TRPM_PZQ_ has emerged as a “polymodal” ion channel, defined as a channel regulated by both chemical as well as physical cues. Chulkov and colleagues demonstrated that membrane stretch activates *Sm*.TRPM_PZQ_, a potentially relevant cue for an aquatic parasite that must traverse a pressurized vascular system [20]. Schistosome contraction is known to be regulated by stretch [75]. As there are a multitude of sensory demands across the parasitic life cycle, other environmental cues may emerge as TRPM_PZQ_ regulators, potentially encompassing both parasite-derived and host-derived ligands [76]. Unbiased screening approaches, as well as biased probing of known vertebrate TRP channel regulators will be of value in identifying such ligands. Elaboration of the TRPM_PZQ_ interactome to define interacting proteins will be another priority to identify other regulators of the channel complex. Unravelling the functional consequences of both ligand and accessory protein regulation of TRPM_PZQ_ will surely reveal new targets for chemotherapeutic attack.

## Conclusions

TRPM_PZQ_ has recently emerged as a druggable parasite ion channel, with the evidence outlined in this review supportive of this ion channel acting as the relevant parasite target of PZQ. Recent work has elaborated the key properties of this ion channel and identified future experimental priorities. The prospect of identifying novel pharmacological tools for probing the function of TRPM_PZQ_, as well as for other ion channels within the parasitic flatworm TRPM subfamily [71] and the broader TRP channel superfamily [77], will further our understanding of the roles of these sensory ion channels throughout the parasitic lifecycle. This is currently a very exciting time for anthelmintic drug development, with new broad spectrum oxamniquine derivatives [78], highly potent antischistosomal chemotypes [79], as well as novel TRPM_PZQ_ activators all recently emerging [32,49]. Hopefully, this will yield exciting advances for treating parasitic flatworm infections within the not-too-distant future.

**Top Five Papers**

1. Andrews P, Thomas H, Pohlke R, Seubert J. Praziquantel. Med Res Rev. 1983;3(2):147–200. Epub 1983/04/01. doi: 10.1002/med.2610030204. PubMed PMID: 6408323.
2. Bais S, Churgin MA, Fang-Yen C, Greenberg RM. Evidence for Novel Pharmacological Sensitivities of Transient Receptor Potential (TRP) Channels in Schistosoma mansoni. PLoS Negl Trop Dis. 2015;9(12):e0004295.
3. Park SK, Gunaratne GS, Chulkov EG, Moehring F, McCusker P, Dosa PI, et al. The anthelmintic drug praziquantel activates a schistosome transient receptor potential channel. J Biol Chem. 2019;294(49):18873–18880.
4. Park SK, Friedrich L, Yahya NA, Rohr CM, Chulkov EG, Maillard D, et al. Mechanism of praziquantel action at a parasitic flatworm ion channel. Sci Transl Med. 2021;13(625):eabj5832. Epub 2021/12/23. doi: 10.1126/scitranslmed.abj5832
5. Le Clec’h W, Chevalier FD, Mattos ACA, Strickland A, Diaz R, McDew-White M, et al. Genetic analysis of praziquantel response in schistosome parasites implicates a transient receptor potential channel. Sci Transl Med. 2021;13(625):eabj9114.

**Key Learning Points**

- Outline the key properties of the parasitic flatworm ion channel, TRPM_PZQ_.
- Collate current evidence supporting TRPM_PZQ_ as the target of the clinical drug, praziquantel.
- Suggest directions for future experimental work.
